# New developments in the pathology of malignant lymphoma. A review of the literature published from January–April 2016

**DOI:** 10.1007/s12308-016-0277-4

**Published:** 2016-06-13

**Authors:** J. Han van Krieken

**Affiliations:** Department of Pathology, Radboud University Medical Centre, P.O. Box 9101, 6500 HB Nijmegen, The Netherlands

## Introduction

Science and knowledge progress rapidly. How to make something out of huge data sets, large amounts of information that comes on a daily base to us through various sources? Although the bias of hypothesis-driven research may indeed prevent to discover the unusual, the downside of it is that just finding significant correlations overflows the literature (see also the editorial in this issue; [1]). This review gives some examples of the different approaches to science, and it is up the reader to draw conclusions.

## Biology of lymphoma

### Hodgkin lymphoma

The NF-kB pathway is activated in several lymphoma types. De Oliveira et al. [2] analyzed the transcriptome of Hodgkin lymphoma (HL) cell lines. They found that various NF-kB subunits are recruited to a large number of genes. Thus, NF-kB up- and downregulates gene sets that are both distinct and overlapping and are associated with diverse biological functions. p50 and p52 are formed through NIK-dependent p105 and p100 precursor processing in HL cells and are the predominant DNA-binding subunits. Logistic regression analyses of combinations of the p50, p52, RelA, and RelB subunits in binding regions that have been assigned to genes they regulate reveal a cross-contribution of p52 and p50 to canonical and non-canonical transcriptomes. These analyses also indicate that the subunit occupancy pattern of NF-kB-binding regions and their distance from the genes they regulate are determinants of gene activation versus repression. The pathway-specific signatures of activated and repressed genes distinguish HL from other NF-kB-associated lymphomas and inversely correlate with gene expression patterns in normal germinal center B cells.

Paydas et al. [3] describe the profile of 377 micro (mi) RNAs in 32 HL cases. A whole series of miRs were differently expressed compared to normal germinal center (GC) B cells, some with higher, others with lower expression, but there was no difference in miRNA profile according to the age, sex, stage, response to treatment, DFS, and OS. The authors conclude that we need more studies evaluating miRNA profile and clinical outcome in HL. My conclusion would be that we need to create better hypothesis before we do analyses that result in very large datasets.

### B cell lymphomas

Gastric extranodal marginal zone lymphoma (ENMZL) is a consequence of *H. Pylori* (HP) infection, but most patients who have HP gastritis will not develop a lymphoma or carcinoma. Gossmann et al. [4] used BALB/c mice with a gain-of-function mutation in the Plcg2 gene (Ali5), critical for B cell maintenance, to analyze its role in the development of gastric ENMZL. Heterozygous BALB/c Plcg2Ali5/+ and wild-type (WT) mice were infected with *Helicobacter felis* (*H. felis*). In contrast to their hypothesis, Plcg2Ali5/+ mice developed ENMZL less frequently than the WT littermates after long-term infection of 16 months. Infected Plcg2Ali5/+ mice showed downregulation of pro-inflammatory cytokines and decreased *H. felis*-specific IgG1 and IgG2a antibody responses. These results suggested a blunted immune response of Plcg2Ali5/+ mice towards *H. felis* infection. Intriguingly, Plcg2Ali5/+ mice harbored higher numbers of CD73 expressing regulatory T cells (Tregs), possibly responsible for impaired immune response towards Helicobacter infection. They suggest that Plcg2Ali5/+ mice may be protected from developing gastric ENMZL as a result of elevated Treg numbers, reduced response to *H. felis*, and decrease of pro-inflammatory cytokines. Of course, it would be interesting to see whether such differences exist between individuals with HP gastritis with and without ENMZL.

Cui et al. [5] investigated human gastric ENMZL with respect to cyclooxygenase-2 (COX-2) and interleukin-32 (IL-32) expression since these have been suggested to be significant in tumor progression and prognosis. COX-2 and IL-32 protein expression was higher in 31 primary gastric B cell lymphoma patients compared to 19 chronic gastritis patients, especially so in patients who had HP-positive lymphomas; the expression level of COX-2 was positively correlated with the expression level of IL-32. Furthermore, COX-2 expression was associated with an aggressive tumor type, higher number of Ki-67 positive cells, lymph node metastasis, and advanced stage. IL-32, and to a lesser degree COX-2, expression was found to be correlated with frequent lymph node metastasis and an advanced stage and poor survival.

Broutier et al. [6] use knowledge form other cancers to study lymphoma and found a promising target for therapy. Deleted in colorectal carcinoma (DCC) constrains tumor progression by inducing apoptosis unless engaged by its ligand netrin-1 in breast and colorectal cancers. Using a transgenic mouse model, they found that inhibition of DCC-induced apoptosis is also associated with lymphomagenesis. In human diffuse large B cell lymphoma (DLBCL), they demonstrate an imbalance of the netrin-1/DCC ratio which suggests a loss of DCC-induced apoptosis, either via a decrease in DCC expression in the GC subtype or by upregulation of netrin-1 in activated B cell (ABC) one. Such imbalance is also observed in mantle cell lymphoma (MCL). Using a netrin-1 interfering antibody, they demonstrate both in vitro and in vivo that netrin-1 acts as a survival factor for ABC-DLBCL and MCL tumor cells. Together, these data suggest that interference with the netrin-1/DCC interaction could represent a promising therapeutic strategy in netrin-1-positive DLBCL and MCL.

High expression of the forkhead box P1 (FOXP1) transcription factor distinguishes the more aggressive ABC-DLBCL subtype from germinal center (GC)-DLBCL subtype and is correlated with poor outcomes. Dekker et al. [7] show that sustained FOXP1 expression is vital for ABC-DLBCL cell line survival. Genome-wide analyses revealed direct and indirect FOXP1 transcriptional enforcement of ABC-DLBCL hallmarks, including the classical NF-kB and MYD88 pathways. FOXP1 promoted gene expression underlying transition of the GCB-cell to the plasmablast—the transient B cell stage targeted in ABC-DLBCL transformation—by antagonizing pathways distinctive of GCB-DLBCL, including that of the GCB “master regulator,” BCL6. Cell line-derived FOXP1 target genes that were highly correlated with FOXP1 expression in primary DLBCL accurately segregated the corresponding clinical subtypes of a large cohort of primary DLBCL isolates and identified conserved pathways associated with ABC-DLBCL.

Hafsi et al. [8] show that available data can be used for new insights. They are interested in the oncogenic transcription factor, Yin Yang 1 (YY1), which has been reported to be overexpressed in several malignancies. A total of 57 miRNAs that are potentially capable of targeting YY1 was identified through in silico approaches. The search of publicly available NHL datasets, including paired mRNA and miRNA data highlighted a significant correlation between the expression levels of YY1 and the expression levels of a limited set of miRNAs. They found it intriguing that, both hsa-miR-363 and hsa-miR-200a belong to the top 20 miRNAs that were found to be downregulated in Burkitt’s lymphoma (BL) tissue compared to normal tissue. Although further validation studies are warranted, the identification of these two miRNAs associated with the upregulation of YY1 in BL may provide further insight into the pathogenesis of this tumor and may contribute to more personalized and targeted treatment approaches for patients with this disease. Also in this case, an approach based on a hypothesis rather than just an interest in a specific protein might result in more relevant data.

### T cell lymphoma

Nairismägi et al. [9] used whole exome sequencing of four cases of type II enteropathy-associated T cell lymphoma (EATL-II) and confirmed the findings in another 42 cases by amplicon-based deep sequencing. STAT5B was mutated in 63 % of cases, JAK3 in 35 % and GNAI2 in 24 %, with the majority occurring at known activating hotspots in key functional domains. Moreover, the STAT5B locus carried copy-neutral loss of heterozygosity resulting in the duplication of the mutant copy, suggesting the importance of mutant STAT5B dosage for the development of EATL-II. Furthermore, they show dysregulation of the JAK-STAT and GPCR pathways by gene expression profiling. In vitro overexpression of GNAI2 mutants led to the upregulation of pERK1/2, a member of MEK-ERK pathway. Notably, inhibitors of both JAK-STAT and MEK-ERK pathways effectively reduced viability of patient-derived primary EATL-II cells, indicating potential therapeutic strategies for this neoplasm with no effective treatment currently available. This fine work is now ready to be tested in the clinic.

A whole other approach was chosen by Hao et al. [10]. Because gain-of-function mutations in isocitrate dehydrogenase 1 (IDH1) can be key drivers of malignancies of the T cell lineage, they analyzed the T cell compartment in a conditional knock-in (KI) mouse model of mutant IDH1 and observed the development of a spontaneous T cell acute lymphoblastic leukemia (T-ALL) in these animals. The disease was transplantable and maintained expression of mutant IDH1. Whole exome sequencing revealed the presence of a spontaneous activating mutation in Notch1, one of the most common mutations in human T-ALL, suggesting IDH1 mutations may have the capacity to cooperate with Notch1 to drive T-ALL. To further investigate the IDH1 mutation as an oncogenic driver in the T cell lineage, we crossed IDH1-KI mice with conditional Trp53 null mice, a well-characterized model of T cell malignancy, and found that T cell lymphomagenesis was accelerated in mice bearing both mutations. It would be interesting to see whether these findings can be confirmed in human cases.

Anaplastic large cell lymphoma (ALCL) is a peripheral T cell lymphoma presenting mostly in children and young adults. Malcolm et al. [11] present a mouse model of ALCL where the malignancy is initiated in early thymocytes, before T cell receptor (TCR) β-rearrangement. They show that a TCR is required for thymic egress and development of peripheral murine tumors, yet this TCR must be downregulated for T cell lymphomagenesis. In keeping with this, clonal TCR rearrangements in human ALCL are predominantly in-frame, but often aberrant, with clonal TCRα but no clonal TCRβ rearrangement, yielding events that would not normally be permissive for survival during thymic development. These results explain indeed the clonality findings in many cases.

## Epidemiology of lymphoma

Perry et al. [12] describe the distribution of non-Hodgkin lymphoma (NHL) subtypes in Southern Africa. Five expert hematopathologists (no definition given) classified 487 consecutive cases of NHL using the World Health Organization classification and compared the results to North America and Western Europe. Southern Africa had a significantly lower proportion of low-grade (LG) (34 %) and a higher proportion of high-grade (HG) B cell lymphoma (B-NHL;52 %) compared to Western Europe (55 and 36 %) and North America (56 and 34 %); BL was more common, 8 versus 2 and 3 %, most likely due to human immunodeficiency virus infection. When the patients were divided by race, whites had a significantly higher frequency of LG (60 %) and a lower frequency of HG B-NHL (33 %) compared to blacks (23 and 63 %), whereas the other races were intermediate; the median ages of whites with LG B-NHL, HG B-NHL, and T-NHL (64, 56, and 67 years) were significantly higher than those of blacks (55, 41, and 34 years). The authors conclude that further epidemiological studies are needed to better understand these differences, but one wonders whether access to health care may be a critical issue here too.

It is now well known that rare cancers are common, but hard to study. O’Suoji et al. [13] used data from the Children’s Oncology Group Rare and Cutaneous NHL registry’s to determine the pathologic, biologic, and clinical features of rare and cutaneous pediatric NHL. In 101 lymphomas, there was a 98 % concordance between the reviewing study pathologists and an 88 % concordance between the central and institutional pathology review, remarkably high for rare lymphomas. Children with pediatric follicular lymphoma (FL), nodal and extranodal primary cutaneous, primary central nervous system lymphoma, and subcutaneous panniculitis-like T cell lymphomas have 100 % survival at a median of 2 years from enrollment. There are early deaths, mostly from progressive disease, in subjects with peripheral T cell (not otherwise specified), NKT, and hepatosplenic T cell lymphomas.

Population-based data are very relevant to get a good idea on the features of rare diseases, since centers will have a selection of patients resulting in a certain bias. Strobbe et al. [14] made use of such a database to describe nodular lymphocyte predominant HL (NLPHL). A disadvantage of the use population-based data is the longer period that patients need to be collected, in this case 20 years, before meaningful conclusions can be taken and over time differences in criteria and treatment occur. It was therefore important that at least the pathology was reviewed. Seventy-three cases of NLPHL were analyzed with a median follow-up of 65 months (range 4–257 months). Median age at diagnosis was 43 years (range 1–87), 85 % of the patients were male, B symptoms were present in 6 %, and stage I/II disease was most common (75 %). Patients were primarily treated with radiotherapy (51 %), chemotherapy (26 %), combined modality (radiotherapy and chemotherapy) (11 %), or surgical excision with careful watch-and-wait (12 %). Relapses occurred in only seven patients (10 %) after a median of 26 months (21–74 months). Six patients (8 %) developed histologic transformation to large cell lymphoma. Five patients (7 %) died during follow-up due to progression of NLPHL (*n* = 1), histologic transformation (*n* = 2), and intercurrent deaths (*n* = 2). The estimated 10-year overall survival was 94.0 % and the 10-year progression-free survival 76 %. A similar study was performed by Kenderian et al. [15] with a focus on transformation, a larger study, but from a center and even covering 40 years. Between 1970 and 2011, 222 consecutive adult patients with new untreated NLPHL were identified. Median age at diagnosis was 40 years and 146 (66 %) were males. The median follow-up was 16 years. Seventeen patients (8 %) developed a transformation to DLBCL, exactly the same percentage as the Strobbe study. The median time to transformation was 35 months (6–268). In a multivariate analysis, use of any prior chemotherapy and splenic involvement were significantly associated with increased risk of transformation. The five-year overall survival in those with transformed disease was 76 % but transformation did not adversely affect overall survival. Both studies confirm the distinct characteristics of NLPHL with a good long-term prognosis. It is likely that it is possible to reduce treatment intensity in early stage NLPHL without affecting long-term outcome, which is especially relevant in young individuals as is common in this disease.

Acquired C1-inhibitor (C1-INH) deficiency (C1-INH-AAE) is a rare condition resulting in acquired angioedema (AAE) and about 33 % of the patients develop NHL. Castelli et al. [16] report the follow-up of 72 C1-INH-AAE patients, followed for a median of 15 years. The median age was 71 years with a median age at onset of angioedema symptoms was 58. Twenty patients were diagnosed with LG non-follicular B-NHL (75 % were splenic MZL), one with FL and MCL each and two with DLBCL. Fifteen NHLs were diagnosed at onset of AAE or thereafter (3 months to 7 years), eight had already been diagnosed at onset of angioedema. Two of 24 patients remain on watchful wait. Thirteen of 24 received chemotherapy; two received rituximab. Three underwent splenectomy. All 18 patients receiving therapy for NHL experienced post-treatment reduction in AAE symptoms. This study indicates that a clonal B cell proliferation is underlying AAE in a major subset of patients and can lead to production of C1-INH-neutralizing autoantibodies. The post-germinal center origin of these B-NHLs suggests that immune stimulation may contribute to the lymphomagenesis.

A subset of DLBCL is CD30 positive. Gong et al. [17] studied 232 cases of de novo DLBCL in East China to investigate the prevalence and clinicopathological features of CD30-positive DLBCL using a panel of immunohistochemical markers. Applying a >0 % threshold (which is quite low in my mind), CD30 was expressed in approximately 12 % of the tumors from patients with Epstein-Barr virus (EBV) negative DLBCL, affecting younger people and showing a lower frequency of BCL2 expression and MYC/BCL2 co-expression. Patients with CD30-positive DLBCLs showed better progression-free survival and overall survival compared with patients with CD30-negative DLBCLs.

## Defining entities

### B cell lymphomas

An interesting hypothesis was put forward in the previous issue of the *Journal of Hematopathology* by van den Brand et al. [18]: quite some cases that are diagnosed as FL but lack a t(14;18) may actually be nodal(N) MZL. They studied 33 low-grade B cell lymphomas without a BCL2 break and compared the results with cases that had a BCL2 break. Follicular colonization was noted in the lymphomas without a *BCL2* rearrangement, which was strongly overlapping with the morphological features of NMZL. This study raises the hypothesis that a subset of LG B-NHL with a follicular growth pattern but without a *BCL2* translocation actually represents NMZL.

Batlle-López et al. [19] used three immunohistochemical approaches to separate GC from ABC-DLBCL on tissue microarrays (TMAs) with samples from 297 patients. In addition, they performed FISH for MYC, BCL2, IRF4, and BCL6. Non-GC-DLBCL patients had significantly worse progression-free survival and overall survival, based on all three (Choi, Visco-Young, and Hans) algorithms, indicating that any of these algorithms would be appropriate for identifying patients who require alternative therapies to R-CHOP. While MYC abnormalities had no impact on clinical outcome in the non-GC subtype, patients with isolated MYC rearrangements and a GC-DLBCL phenotype had worse survival and therefore might benefit from more aggressive treatment approaches.

Lu et al. [20] approached the same issue and analyzed the antibodies applied in the Hans algorithm and other genetic factors in 601 DLBCL patients and the prognostic value of the Hans algorithm in 306 cases who were treated with chemoimmunotherapy. Patients with GC subtype indeed have better overall survival and progression-free survival than non-GC cases. However, CD10- and MUM1-positive cases and cases that were negative for CD10, BCL6, and MUM, showed different clinical characteristics and prognosis to others that were assigned to the same cell-of-origin group, indicating that the story is not complete, and calls for a consensus meeting on the use of immunohistochemistry to determine DLBCL subgroups.

Roth et al. [21] describe the flow cytometry results of 20 “double” or “triple” hit lymphomas (D/THL) with recurrent translocations involving MYC and BCL2 and/or BCL. Most (89 %, 17/19) D/THL were CD10(+), 47 % (9/19) lacked surface light chain, and a significant subset had low expression of CD45 (47 %, 9/19), CD20 (42 % 8/19), and/or CD19 (39 %, 7/18), which did not vary by genetic subgroup. However, compared to B-lymphoblastic lymphoma (LBL), D/THL less frequently underexpressed CD45 and CD20. Lower levels of BCL2 expression were noted in the BCL6(+)/MYC(+) and BCL2(+)/BCL6(+)/MYC(+) subgroups versus BCL2(+)/MYC(+) cases. Dim CD45 expression correlated with inferior survival. The authors conclude that although there is some overlap with B-LBL, D/THL demonstrates a characteristic immunophenotype which may have prognostic significance. The data also show that flow cytometry cannot replace genetic testing in these cases.

Moench et al. [22] investigated the same tumor types and report clinicopathologic features of 13 cases (nine DHL/four THL). The median age was 59 years (range 30–74) and patients included eight females and five males. Presentation included enlarging lymphadenopathy/masses (11 patients) and abnormal peripheral blood findings (two patients). Features which raised the differential of an immature neoplasm included terminal deoxynucleotidyl transferase positivity (four cases, two THL/two DHL); dim CD45 expression (seven cases), lack of CD20 (two cases), or lack of surface immunoglobulin light chain (three cases) by flow cytometry; and blastoid morphology (two cases). They conclude that expression of TdT in a B cell lymphoma with mature features or expression of surface light chain in a case otherwise suggestive of B-lymphoblastic leukemia/lymphoma should initiate investigations to exclude DHL/THL. This raises the question, what is the optimal workup nowadays for a DLBCL, an issue that can be addressed in the afore proposed consensus meeting.

### T cell lymphomas

Tanaka et al. [23] investigated the expression of TCRβ and TCRγ protein expression of 42 gastrointestinal T cell lymphomas. Nine (21 %) were positive for TCRγ protein expression and five of these expressed TCRβ as well. TCRβ positivity without TCRγ expression was seen in nine cases (21 %). Twenty-four patients (57 %) were negative for both TCRβ and γ. TCRγ cases were characterized by exclusive involvement of intestinal sites (100 % vs. 11 %), but not of the stomach (0 % vs. 78 %). Furthermore, TCRγ positivity was an independent unfavorable prognostic factor. According to the authors, this indicates that intestinal γδ T cell lymphoma constitutes a distinct disease entity.

The splicing factor neuro-oncological ventral antigen 1 (NOVA1) is present in T cells of tertiary lymphoid structures. Kim et al. [24] found that tumor cells of T and NK-cell lymphomas showed higher expression levels of NOVA1 than normal paracortical T cells, and 57 % of 177 T and NK-cell lymphoma cases had diffuse and strong expression. The NOVA1 expression level varied according to the subtype; it was higher in angioimmunoblastic T cell lymphoma (AILT), anaplastic lymphoma kinase (ALK)-negative anaplastic large cell lymphoma (ALCL), and T-LBL, but it was lower in ALK-positive ALCL. In almost all B cell lymphomas, NOVA1 expression was very low or negative. They conclude that upregulated NOVA1 expression seems to be a specific biological feature of activated T cells and T and NK-cell lymphomas.

Dobashi et al. [25] investigated extranodal natural killer/T cell lymphoma (ENKTL) for mutations in the JAK-STAT pathway, recently reported in ENKTL cases. Targeted capture sequencing of 602 cancer-related genes from 25 frozen ENKTL samples revealed recurrent somatic mutations involving BCOR (32 %), TP53 (16 %), DDX3X (12 %), FAT4 (8 %), NRAS (8 %), MLL3 (12 %), and MIR17HG (8 %). The pattern of BCOR aberrations (one nonsense and five frame-shift mutations, a mutation leading to a splicing error, and gene loss) suggested that loss of function of BCOR was the functionally important outcome of such changes. The literature was reviewed and the public data on BCOR aberrations was reanalyzed and it was found that the aberrations were frequently found in myeloid neoplasms, but, interestingly, were highly specific to ENKTL among lymphoid malignancies. Given the high frequency and pattern of aberration, BCOR is likely to play an important role in ENKTL pathogenesis as a tumor suppressor gene.

Adult T cell leukemia/lymphoma (ATLL) is a rare T cell neoplasm caused by human T cell leukemia virus type 1 occurring in specific regions on the world. Tobayashi et al. [26] analyzed 184 cases of peripheral T cell lymphoma, including 113 cases of ATLL for mutations in CCR4. These were present in 27 % (30/113) of cases of ATLL and 9 % (4/44) of cases of peripheral T cell lymphoma not otherwise specified. Identified mutations included nonsense (NS) and frame-shift (FS) mutations. There were no differences in clinicopathological features between ATLL cases with and without CCR4 mutation. All ATLL cases with CCR4 mutations expressed CCR4 with higher CCR4 expression in cases with NS mutations than in cases with wild-type (WT) CCR4. Furthermore, among ATLL cases, FS mutation was associated with a poor prognosis, compared with NS mutation and WT CCR4. These results indicate that CCR4 mutation is an important determinant of the clinical course in ATLL, and that NS and FS mutations of CCR4 have a different effect in the pathogenesis.

### Cutaneous lymphomas

Lee et al. [27] investigated clinical features of 52 primary and secondary cutaneous ALCL. Although skin lesion characteristics did not significantly differ between groups, the head and neck location was more common in primary cutaneous ALCL, whereas cutaneous lesion extent was greater in secondary cutaneous ALCL. Skin lesion extent in primary cutaneous ALCL was indicative of extracutaneous dissemination development and skin lesion relapse. Neither ALK expression nor clinical stage affected skin lesion characteristics in secondary cutaneous ALCL. Patients with primary cutaneous ALCL demonstrated better survival outcomes. The skin lesion extent and location on the leg were associated with a tendency towards a poorer prognosis in primary cutaneous ALCL. The secondary cutaneous ALCL prognosis was not influenced by skin lesion characteristics.

The diagnosis of panniculitis-like T cell lymphoma (SPTCL) can be difficult. Especially, cases of SPTCL and lupus erythematosus panniculitis (LEP) can have clinical and histopathologic overlap, raising the possibility that they represent opposite ends of a disease spectrum. SPTCL, however, is typically associated with greater morbidity and risk for hemophagocytic lymphohistiocytosis (HLH). LeBlanc et al. [28] present their experience regarding the histopathologic, immunophenotypic, and molecular findings of 13 patients with SPTCL and seven with LEP. Six SPTCL patients developed HLH, including two who were under the age of 21 years. In the SPTCL group, 2 of 13 patients died of disease. In contrast, there was no mortality or development of HLH in the LEP cohort. With a limited panel (Ki-67, CD3, CD4, and CD8 immunostains) foci of “Ki-67 hotspots” were present among cytotoxic atypical CD8+ T cells in SPTCL. Ki-67 hotspots were not identified in LEP, thus aiding the distinction of SPTCL from LEP. Lymphocyte atypia combined with adipocyte rimming of CD8+ T cells within Ki-67 hotspots was also highly specific for the diagnosis of SPTCL. Hyaline lipomembranous change, B cell aggregates, plasmacytoid dendritic cell clusters, and plasma cell aggregates favored the diagnosis of LEP but were identified in some cases of SPTCL as well including patients with HLH. Especially the Ki67 hot spots idea is new and can be helpful in this situation.

## New entities/subtypes

Lorenzi et al. [29] report the clinical, morphological, phenotypical, and molecular features of three cases of a hitherto unreported variant of Epstein-Barr virus (EBV)-positive, human herpes virus 8 (HHV8)-negative large B cell lymphoma with exclusive intrafollicular localization. These cases occurred in elderly individuals (63, 77, and 65 years old; one male, two females) without obvious immune-deficiency, who presented with high stage disease. The lymph nodes showed an effaced nodular architecture with abnormal B cell follicles colonized by EBV+ large, pleomorphic atypical cells, including Reed-Sternberg-like cells, showing an activated B cell phenotype (CD10-FOXP1-BCL6-IRF4+ or CD10-FOXP1+BCL6+IRF4+) and intense expression of CD30. No monoclonal light-chain restriction was detected by immunohistochemistry or in situ hybridization, and IGH rearrangement was polyclonal; notably, EBV clonality was present in one case. Lymphoma cells in all cases showed diffuse expression of the Myc protein, while Bcl2 was dim or negative; moreover, the strong expression of phosphorylated-STAT3 in tumor cell nuclei suggested activation of the JAK-STAT pathway. FISH analysis was performed in two cases and showed no translocations of BCL2, BCL6, MYC, and PAX5 genes. Response to treatment was poor in 2/3 patients: one died after 18 months and one is alive with disease after 12 months. This report on intrafollicular EBV-positive large B cell lymphoma expands the spectrum of EBV-associated lymphoproliferative disorders in immunocompetent individuals.

Initially MYD88 mutations were seen as typical for lymphoplasmacytic lymphoma (LPL) but the spectrum has expanded. Rovira et al. [30] describe the incidence, clinicopathological features, and outcome of 213 patients (115 M/98 F; median age, 65 years) with DLBCL treated with immunochemotherapy in a single institution with respect to MYD88 mutational status. MYD88 mutations were found in 47 cases (22 %), including L265P in 39 and S219C and M232F in four cases, respectively. Patients with MYD88 L265P were older, presenting frequent extranodal involvement, and mostly corresponded to the ABC subtype. Five-year overall survival for MYD88 wild-type, MYD88 L265P, and other variants was 62, 52, and 75, respectively. However, the prognostic impact was lost when the ABC subtype was taken into account. The data are in line with those from Taniguchi et al. [31] who analyzed primary breast (PB)-DLBCL, which comprises <3 % of extranodal lymphomas. It frequently reveals an ABC phenotype and it has been reported to have gain-of-function mutations in MYD88, CD79B, CARD11, and TNFAIP3, resulting in constitutive activation of the NF-kB pathway. Using Sanger sequencing on 46 breast DLBCL cases, they found MYD88 L265P and CD79B mutations in 27/46 (59 %) and 11/33 (33 %) cases, respectively. Twenty-eight of 46 cases met the criteria for PB-DLBCL. The frequency of MYD88 L265P and CD79B mutations was 16/28 (57 %) and 9/23 (39 %), respectively, in PB-DLBCL and 11/18 (61 %) and 2/10 (20 %), respectively, in the disseminated cases presenting in the breast, which might suggest that these belong to the same disease entity.

Most of the post-transplant lymphoproliferative diseases (PTLD) are EBV driven, but a subset is EBV negative. Finalet Ferreiro et al. [32] performed array-comparative genome hybridization (aCGH) analysis of 21 EBV(+), 6 EBV(−) PTLD with morphology of DLBCL, and 11 control DLBCL, and combined genomic data with their previously published transcriptomic data. The analysis showed that EBV(+) and EBV(−) cases have distinct aCGH profiles and shared only one recurrent imbalance. EBV(−) displayed at least ten aberrations recurrent in DLBCL, among which characteristic gain of 3/3q and 18q, and loss of 6q23/TNFAIP3 as well as 9p21/CDKN2A. The most prevalent aberration in EBV(+) cases was gain/amplification of 9p24.1 targeting PDCD1LG2/PDL2. These data indicate that the FOXP1 oncogene and the tumor suppressor CDKNA2 implicated in EBV(−) DLBCL, do not play a role in the pathogenesis of EBV(+) PT-DLBCL. Altogether, genomic profiling of PT-/IC-DLBCL confirms that EBV(−) and EBV(+) PT-DLBCL are distinct entities, while EBV(−) PT-DLBCL has features in common with DLBCL, however EBV-positive DLBCL outside the transplant setting were not included.

## Pitfalls in lymphoma diagnosis

As I wrote in my editorial [1], we know now that cells with genetic alterations may occur in healthy individuals, and of course these cells can expand to some extent. The border between a benign expansion and a malignant process is there probably impossible to define. A criterion we use in general is expansion beyond the normal anatomical structure. It is therefore interesting to see what the consequences are of so-called FL and MCL in situ (FLIS and MCLIS), because these are accumulations of cells with a genetic alteration but still in their normal microenvironment. Bermudez et al. [33] studied a series of 341 consecutive lymph node resection specimens from patients diagnosed with colorectal (201 cases) and breast (140 cases) adenocarcinoma between 1998 and 2000. Incidental and isolated FLIS was identified in 11/341 patients (3 %), whereas incidental and isolated MCLIS was found in 2/341 patients (1 %). None of these patients developed overt lymphoma, which fits with the notion that these represent incidental findings for which probably the term lymphoma should be avoided altogether. Next, a second series of five cases with incidental and isolated FLIS was identified from consultation files and also none of these patients developed overt lymphoma. Nybakken et al. [34] addressed a similar question, what is the clinical meaning of the presence of isolated follicles that exhibit atypical morphologic features? They collected seven cases with centroblast-predominant isolated follicles and absent BCL2 staining in otherwise-normal lymph nodes. Four of these showed a clonal B cell proliferation amid a polyclonal B cell background (not so surprising, since germinal centers are often (oligo)clonal) all cases lacked the IGH-BCL2 translocation and BCL2 protein expression. Although three patients had invasive breast carcinoma at other sites, none were associated with systemic lymphoma up to 44 months after diagnosis. This work also confirms the importance of architectural changes when a diagnosis of lymphoma is considered. In line with this is the work of van den Brand et al. [35] who present an interesting case in which morphologically there was a FL, but a part of the follicles was BCL2 negative and another part was positive. They were able to demonstrate that both parts belong to the same clone, but due to ongoing mutation, the epitope recognized by the BCL2 antibody was lost in the negative part of the lymphoma. All three articles show the importance of combining morphology and immunophenotype with genetics is important to come to a right diagnosis.

Jain et al. [36] incidentally found a case of ALK-positive ALCL that stained for Napsin A, a marker often used to classify a tumor a lung primary. They stained two other cases from their archive and found the same result and thus they conclude that ALK-positive DLBCL should be considered in the differential diagnosis when evaluating a Napsin A-positive tumor of poorly differentiated morphology and of unknown primary.

## Prognostic factors in lymphoma

Ki-67 has been shown to be a good prognostic marker in MCL. Hoster et al. [37] compared its value with other histological factors like tumor cell morphology and growth pattern in more than 500 cases. Blastoid cytology was associated with inferior survival but not independently of the Ki-67 index. Growth pattern was not independently prognostic. Choi et al. [38] determined semi quantitatively the level of CD20 expression in 48 cases of DLBCL and found a subgroup of the patients with CD20 expression levels below the cut-off score having poor clinical outcome. Yabe at al [39] evaluated 28 cases of hepatosplenic T cell lymphoma (HSTCL) patients to determine factors that may be associated with outcome. The HSTCL cells expressed γδ T cell receptor (TCR) in 20 (74 %), αβ TCR in five (19 %), and neither in two (7 %) patients (one case not assessed). Serum bilirubin level ≥1.5 mg/dL, αβ TCR expression, and trisomy 8 each correlated significantly with shorter survival.

All in all, the briefest paragraph on prognostic factors in the series of review, you can read the argumentation in the previous issue [40].

## Staging

Bone marrow biopsies are slowly being replaced by other methods for staging. Cho et al. [41] investigated pretreatment BM samples of 394 DLBCL patients with clonality testing in addition to microscopic examination. Monoclonal immunoglobulin gene rearrangement was detected in 25 % of cases. Histologic B cell aggregates with the features of large B cell lymphoma aggregates, small cell B cell lymphoma aggregates, or B cell aggregates of unknown biological potential were observed in 12 % of cases (7, 1, and 4 %, respectively). Histologic B cell aggregates were more associated with monoclonality than polyclonality. Cases with both monoclonality and histologic B cell aggregates demonstrated close association with poor prognostic factors such as a higher International Prognostic Index score and showed an inferior overall survival rate when compared to cases with only monoclonality or only histologic B cell aggregates. From their findings, a combination of monoclonality and histologic B cell aggregates within the bone marrow was highly associated with poor prognosis and could be used to determine high-risk DLBLC patients with greater sensitivity and specificity than conventional microscopic examination or immunoglobulin gene rearrangement study alone. A combination with clonality testing in the primary tumor would have been helpful, and a two-step approach might be sensible: only clonality testing in equivocal cases; however, the value of that approach needs to be determined.

Di Martino et al. [42] compared the levels of BCL-1/JH fusion products detected by q-PCR in the concurrent peripheral blood (PB) and bone marrow (BM) aspirate samples from seven patients with MCL. In patients with moderate to high levels of BCL-1/JH copies, the results of q-PCR analysis of PB and BM aspirate samples correlate well. In patients with high levels of BCL-1/JH copies, instead, PB levels are a good indication of tumor burden. Finally, in patients with low levels of BCL-1/JH copies, the t(11;14) may be detected by identification of neoplastic cells. They conclude that their data suggest that PB can be reliably used in place of BM aspirate both for detection of translocation status during minimal residual disease monitoring and for a possible molecular relapse, especially in those patients who have moderate to high levels of BCL-1/JH copies. If these results will be confirmed on a wider number of MCL patients (seven is indeed very few!!), this might be a promising approach.

## Ancillary techniques

Sometimes a complete new technique that is simple but really different comes up and it is interesting to see if the promise becomes real (remember AGNORs…..). Aesif et al. [43] describe a method that separates benign from malignant cells, a holy grail in pathology of course. They developed a staining technique that enables visualization of tissue thiols in situ using bright field microscopy and validated it using gastrointestinal tissue specimens. They used this technique to assess benign tonsillectomy and DLBCL. Tonsillectomy specimens exhibited diffuse presence of free thiols. Staining for reversibly oxidized thiols was confined to germinal center macrophages and sinus histiocytes. DLBCL was strongly positive for free thiols within malignant cells. Finally, in contrast to benign B cells, the malignant cells demonstrated pronounced and diffuse staining for reversibly oxidized thiols, according to the authors, they demonstrated intrinsic differences between benign and malignant cells. I am curious to read the follow-up studies.

It is now well demonstrated that free circulating DNA is partially derived from neoplastic cells and contains at least part of the genetic alterations present in the tumor. Camus et al. [44] developed digital PCR (dPCR) assays for the detection of exportin-1 (XPO1) E571K, EZH2 Y641N, and MYD88 L265P mutations in DLBCL patients, in order to identify patients most likely to benefit from targeted therapies. They demonstrated that these dPCR assays were sufficiently sensitive to detect rare XPO1, EZH2, and MYD88 mutations in plasma cfDNA, with a sensitivity of 0.05 %. This is therefore a promising technique for the management of DLBCL.

Lee et al. [45] used the BIOMED-2 multiplex polymerase chain reaction (PCR) assay in 27 patients using formalin-fixed paraffin-embedded tissues, with subsequent cloning and sequencing of the amplified Ig genes in 17 patients. All 27 cases of primary and corresponding relapsed tumors showed monoclonal rearrangements of the Ig genes. Whereas IgVH or IgVK fragment lengths were identical in 8/27 pairs (30 %), fragment lengths differed in 19/27 pairs (70 %). In 17 cases analyzed by sequencing, an identical VDJ gene rearrangement was confirmed in 4/4 pairs (100 %) with the same fragment lengths and in 10/13 pairs (77 %) with different fragment lengths. Four of 17 primary lymphomas had multiple VDJ rearrangements, and three of them showed an unrelated relapse. Unrelated relapse was observed in 1/8 mantle cell lymphomas, 1/5 diffuse large B cell lymphomas, and a large B cell lymphoma developed in a patient with a small lymphocytic lymphoma. Unrelated relapses developed after a longer disease-free interval and tended to show poorer outcome compared with related relapse. In summary, relapse of a lymphoma from an unrelated clone is uncommon, but can occur in B cell lymphomas. Clonal relationships should be determined by sequencing of the Ig genes, and not just by comparing the PCR product size. However, according to Jiang et al. [46], the situation is more complicated than that. They created a VDJ-sequencing protocol to trace the clonal evolution patterns of DLBCL relapse by exploiting VDJ recombination and somatic hypermutation (SHM), two unique features of B cell lymphomas. When applying this analysis to several diagnosis-relapse pairs, they discovered key evidence that multiple distinctive tumor evolutionary patterns could lead to DLBCL relapse, obviously an area that needs further investigations.

Dubois et al. [47] describe their approach to determine targets for treatment in lymphomas. Their “Lymphopanel” was designed to identify mutations in 34 genes, selected according to literature data and a whole exome sequencing study of relapsed/refractory DLBCL patients. The tumor DNA of 215 patients with CD20+ de novo DLBCL was used and was informative for 96 % of patients. A clear depiction of DLBCL subtype molecular heterogeneity was uncovered with the Lymphopanel, confirming that ABC, GC, and primary mediastinal B cell lymphoma (PMBL) are frequently affected by mutations in NF-kB, epigenetic, and JAK-STAT pathways, respectively. Novel truncating immunity pathway, ITPKB, MFHAS1, and XPO1 mutations were identified as highly enriched in PMBL. Notably, TNFAIP3 and GNA13 mutations in ABC patients treated with R-CHOP were associated with significantly less favorable prognoses. Although this is a very relevant approach, the search for more functional methods to assess sensitivity for specific drugs is badly needed. Chapuy et al. [48] generated and characterized a panel of DLBCL patient-derived xenograft (PDX) models including eight that reflect the immunophenotypic, transcriptional, genetic, and functional heterogeneity of primary DLBCL and one that is a plasmablastic lymphoma. Six of the eight DLBCL models were ABC-type tumors that exhibited ABC-associated mutations such as MYD88, CD79B, CARD11, and PIM1. The remaining two DLBCL PDX models were GCB-type, with characteristic alterations of GNA13, CREBBP, and EZH2, and chromosomal translocations involving IgH and either BCL2 or MYC. Only 25 % (2/8) of the DLBCL PDX models harbored inactivating TP53 mutations whereas 75 % (6/8) of tumors exhibited copy number alterations of TP53 or its upstream modifier, CDKN2A, consistent with the reported incidence and type of p53 pathway alterations in primary DLBCL. By CCC criteria, 6/8 DLBCL PDX models were BCR-type tumors that exhibited selective sIg expression and sensitivity to entospletinib, a recently developed SYK inhibitor. Thus, this group has established and characterized PDX models of DLBCL and demonstrated their usefulness in functional analyses of proximal BCR pathway inhibition.

Gene alterations are important, but proteins do the work. Wu et al. [49] applied two dimensional gel electrophoresis to compare seven GC NHL cell lines with a lymphoblastoid cell line (LCL). An average of 130 spots was at least two folds different in intensity between NHL cell lines and the LCL. They selected approximately 38 protein spots per NHL cell line and linked them to 145 unique spots based on the location in the gel. Thirty-four spots that were found altered in at least three NHL cell lines when compared to LCL were submitted for mass spectography. This resulted in 28 unique proteins, a substantial proportion of these proteins were involved in cell motility and cell metabolism. Loss of expression of B2M and gain of expression of PRDX1 and PPIA was confirmed in the cell lines and in primary lymphoma tissue. Moreover, inhibition of PPIA with cyclosporine A blocked cell growth of the cell lines. This is therefore a promising new approach.

And finally, stop grading FL by eye, use FLAGS [50]!
